# ﻿Magnifying the hotspot: descriptions of nine new species of many-plumed moths (Lepidoptera, Alucitidae), with an identification key to all species known from Cameroon

**DOI:** 10.3897/zookeys.1193.111544

**Published:** 2024-02-28

**Authors:** Peter Ustjuzhanin, Vasily Kovtunovich, Sylvain Delabye, Vincent Maicher, Szabolcs Sáfián, Alexander Streltzov, Robert Tropek

**Affiliations:** 1 Altai State University, Lenina 61, Barnaul, RU-656049, Russia Altai State University Russia Barnaul Russia; 2 Biological Institute, Tomsk State University, Lenina Prospekt 36, Tomsk 634050, Russia Tomsk State University Tomsk Russia; 3 Moscow Society of Nature Explorers, Moscow, Russia Moscow Society of Nature Explorers Moscow Russia; 4 Department of Ecology, Faculty of Science, Charles University, Viničná 7, CZ-12843 Prague, Czech Republic Institute of Entomology, Biology Centre of the Czech Academy of Sciences České Budějovice Czech Republic; 5 Institute of Entomology, Biology Centre of the Czech Academy of Sciences, Branišovská 31, CZ-37005 České Budějovice, Czech Republic Charles University Prague Czech Republic; 6 The Nature Conservancy Gabon, Impasse Edowangani, Libreville, Gabon The Nature Conservancy Gabon Libreville Gabon; 7 Hungarian Natural History Museum, Department of Zoology, Baross utca 13, H-1088 Budapest, Hungary Hungarian Natural History Museum Budapest Hungary; 8 Herzen State Pedagogical University of Russia, 48, Moika Emb., Saint-Petersburg, 191186, Russia Herzen State Pedagogical University of Russia Saint-Petersburg Russia

**Keywords:** Afrotropics, *Alucita*, biodiversity, Cameroon, endemic, microlepidoptera, taxonomy, tropical rainforest

## Abstract

This study confirms Mount Cameroon as an unprecedented hotspot for the diversity of many-plumed moths, with the discovery and description of nine new species: *Alucitafako* Ustjuzhanin & Kovtunovich, **sp. nov.**, *Alucitapyrczi* Ustjuzhanin & Kovtunovich, **sp. nov.**, *Alucitasroczki* Ustjuzhanin & Kovtunovich, **sp. nov.**, *Alucitapotockyi* Ustjuzhanin & Kovtunovich, **sp. nov.**, *Alucitasedlaceki* Ustjuzhanin & Kovtunovich, **sp. nov.**, *Alucitatonda* Ustjuzhanin & Kovtunovich, **sp. nov.**, *Alucitaerzayi* Ustjuzhanin & Kovtunovich, **sp. nov.**, *Alucitasokolovi* Ustjuzhanin & Kovtunovich, **sp. nov.**, and *Alucitahirsuta* Ustjuzhanin & Kovtunovich, **sp. nov**. Additionally, four additional species are reported from the Mount Cameroon area as new for the country: *Alucitaagassizi*, *Alucitadohertyi*, *Alucitaplumigera*, and *Alucitarhaptica*. Of the 89 Alucitidae known from the Afrotropics, the studied area hosts 36 species, most of which are endemic to the area. This unprecedented level of diversity and endemism within this lepidopteran family highlights Mount Cameroon’s significance as a stronghold for specialised insect taxa. Efficient conservation efforts are necessary to protect these ecosystems and their associated unique microlepidopteran diversity.

## ﻿Introduction

Mount Cameroon represents a well-documented hotspot of diversity for many-plumed moths (Lepidoptera, Alucitidae), a group of moths distinguished by the division of their wings into six lobes. In our previous studies (Kovtunovich and Ustjuzhanin 2016; Ustjuzhanin et al. 2018a, 2020a), we described 17 new species of many-plumed moths and reported additional six species within the Mount Cameroon area. By these numbers, Mount Cameroon was revealed as a key hotspot for the group diversity as it hosts a considerable proportion of the 80 species of Alucitidae known from the Afrotropical region prior to this study (De Prins and De Prins 2023).

In this study, we report the remaining material of Alucitidae gathered during our extensive sampling in the Mount Cameroon area between 2014 and 2017 as part of a large ecological project (e.g., Maicher et al. 2020a, b). Nine species of *Alucita* are described as new for science, and four additional species are reported as new for Cameroon. Furthermore, we furnish an identification key for the majority of species (excluding four species with unknown males) reported from Cameroon.

## ﻿Materials and methods

Our sampling of Alucitidae was performed in nine rainforest localities situated on the south-western and southern slopes of Mount Cameroon, spanning from November 2014 to October 2017. The sampled elevations ranged from 30 to 2200 m a.s.l. These diverse localities provided a comprehensive range of regionally available rainforest habitats. All reported specimens were attracted to light. A comprehensive sampling protocol was previously outlined in Ustjuzhanin et al. (2018a) and Maicher et al. (2020a).

Holotypes will be housed in the Nature Education Centre, Jagiellonian University, Kraków, Poland (NECJU), while paratypes and other specimens will be divided between NECJU and the personal collections of P. Ustjuzhanin and V. Kovtunovich, located in Novosibirsk and Moscow, Russia (CUK).

For identification, we dissected and examined genitalia of most specimens, adhering to the established protocol described in Ustjuzhanin et al. (2018a). Each permanent preparation received a unique code that allows for convenient retrieval and cross-referencing in the collections where they are stored. The relevant codes are provided in the captions of the genitalia figures.

The sampling localities are listed below in an alphabetic order:

**Bamboo Camp**. Bamboo Camp (350 m a.s.l.), Mount Cameroon (SW slope), 4.0879°N, 9.0505°E; a lowland rainforest with historical disturbances from selective logging.

**Bimbia-Bonadikombo**. Mexico Camp (30 m a.s.l.), Bimbia-Bonadikombo Community Forest, 3.9818°N, 9.2625°E; a littoral forest in the part of the community forest that is officially disturbance-free, but with extensive current logging (Ferenc et al., 2018).

**Crater Lake**. Crater Lake camp (1450 m a.s.l.), Mount Cameroon (SW slope), 4.1443°N, 9.0717°E; a submontane rainforest locally disturbed by forest elephants.

**Drink Gari**. Drink Gari camp (650 m a.s.l.; also known as “Drinking Gari” or “Drink Garri”), Mount Cameroon (SW slope), 4.1014°N, 9.0610°E; a lowland rainforest with a dense canopy layer.

**Ekonjo**. Ekonjo camp (1150 m a.s.l.), Mount Cameroon (S slope), 4.0881°N, 9.1168°E; an upland closed-canopy rainforest.

**Elephant Camp**. Elephant Camp (1850 m a.s.l.), Mount Cameroon (SW slope), 4.1170°N, 9.0729°E; a montane forest with a sparse canopy layer as a consequence of natural disturbances by forest elephants.

**Mann’s Spring**. Mann’s Spring camp (2200 m a.s.l.), Mount Cameroon (SW slope), 4.1428°N, 9.1225°E; a montane forest at the natural timberline.

**Mapanja**. Mapanja camp (1850 m a.s.l.), Mount Cameroon (S slope), 4.1157°N, 9.1315°E; a montane forest with mostly closed canopy layer.

**PlanteCam**. PlanteCam camp (1100 m a.s.l.; also misspelled as “Planticamp”), Mount Cameroon (SW slope), 4.1175°N, 9.0709°E; an upland rainforest in the transition between the lowland and montane zones, with a sparse canopy layer as a consequence of natural disturbance by forest elephants (Maicher et al. 2020b).

## ﻿Results

### ﻿Descriptions of the new species

#### ﻿ Alucita pyrczi

Taxon classificationAnimaliaLepidopteraAlucitidae

Ustjuzhanin & Kovtunovich sp. nov.

7A5FA868-63C9-5D3E-9F8E-79BB03757A47

https://zoobank.org/46B56C3C-9FD0-4C42-B2A3-D3B6FB64F72A

Figs 1 , 2

##### Type material.

***Holotype*** • ♀, (NECJU 230701), Cameroon, Bamboo Camp, 350 m a.s.l., Mount Cameroon, 4.0879°N, 9.0505°E, 12–20.XII.2014, lgt. V. Maicher, Sz. Sáfián, Š. Janeček, R. Tropek.

##### Differential diagnosis.

Externally, *Alucitapyrczi* closely resembles *Alucitalidiya* Ustjuzhanin & Kovtunovich, 2018 (known so far only from the male, also collected at Bamboo Camp) but can be distinguished by the presence of a pale orange medial band on its hind wings, the orange band on its fore wings being half as wide, and a larger wingspan. In terms of female genitalia, *A.pyrczi* shares similarities in the structure of antrum and the shape of papillae anales and apophyses with *Alucitamolliflua* (Meyrick, 1927) (Figs 24, 25). However, these species are clearly differentiated by the structure of the ductus, the position of the ductus seminalis, and the absence of signa in the bursa copulatrix.

**Figures 1, 2.**
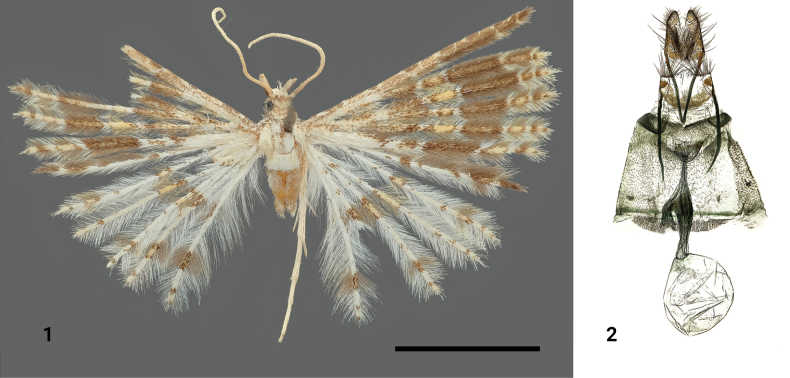
*Alucitapyrczi* Ustjuzhanin & Kovtunovich, sp. nov. **1** adult female, holotype, NECJU**2** female genitalia, holotype, NECJU, preparation slide no. 230701. Scale bar: 5 mm.

##### External characters.

The head and thorax are covered with appressed dark-grey scales, while the tegulae appear white. Labial palpi are dark grey on the outside, with white scales on the inner side, and are twice as long as the longitudinal eye diameter. The third segment is short and white, with scattered tiny brown scales. The antenna is yellowish white. The wingspan measures 18 mm. The fore wing is brown with a distinct orange medium band and is lightened with yellowish white scales at the base. The hind wings are noticeably paler than the forewings and feature a pale orange medial band. The lobes of the hind wings have dark-brown and orange spots of scales submarginally. The fringes on the hind wings are whitish apart from sections of dark-brown hairs around the medial band and submarginal spots, and the distal half of the costa of the first lobe of the hindwing is dark brown. The hind legs are pale-yellow.

##### Female genitalia.

The papillae anales are narrow and elongated. The posterior apophyses are shorter than the anterior apophyses. The antrum is wide and goblet-like, sclerotised. The ductus between the ductus seminalis and antrum is narrow and short. The ductus widens significantly at the junction with the ductus seminalis and narrows at its entrance to the bursa copulatrix. The bursa copulatrix is rounded, and no signa are present.

##### Distribution.

The species was found in Cameroon only.

##### Flight period.

The species was collected in December.

##### Etymology.

The species is named in honour of the Polish lepidopterist Tomasz Wilhelm Pyrcz, who contributed significantly to the collection and study of butterflies and moths in many parts of the world, including Cameroon.

#### ﻿ Alucita sroczki

Taxon classificationAnimaliaLepidopteraAlucitidae

Ustjuzhanin & Kovtunovich sp. nov.

83456EF2-23A9-5F7A-AEC2-1738DE37682D

https://zoobank.org/294E0CD2-7B73-41F5-B048-CCB7D09CC210

Figs 3–5

##### Type material.

***Holotype*** • ♂, (NECJU 230702), Cameroon, Bamboo Camp, 350 m a.s.l., Mount Cameroon, 4.0879°N, 9.0505°E, 17–23.IV.2015, lgt. V. Maicher, Sz. Sáfián, Š. Janeček, R. Tropek. ***Paratypes*** • 4 ex., (NECJU, CUK) same data as holotype • 3 ex., (NECJU, CUK), 11–23.IV.2015, same data as holotype • 5 ex., (NECJU, CUK), Cameroon, PlanteCam, 1100 m a.s.l., Mount Cameroon, 4.1175°N, 9.0709°E, 09–14.IV.2015, lgt. V. Maicher, Sz. Sáfián, Š. Janeček, R. Tropek • 7 ex., (NECJU, CUK), Cameroon, Crater Lake, 1500 m a.s.l., Mount Cameroon, 4.1443°N, 9.0717°E, 23–29.IV.2017, lgt. V. Maicher, P. Potocký, S. Delabye • 8 ex., (NECJU, CUK), 17–25.II.2017, lgt. P. Potocký, Sz. Sáfián, J. Mertens, Š. Janeček, R. Tropek • 1 ♂, (CUK), Cameroon, Mapanja, 1850 m a.s.l., Mount Cameroon, 4.1157°N, 9.1315°E, 23–28.X.2017, lgt. V. Maicher, S. Delabye.

**Figures 3–5.**
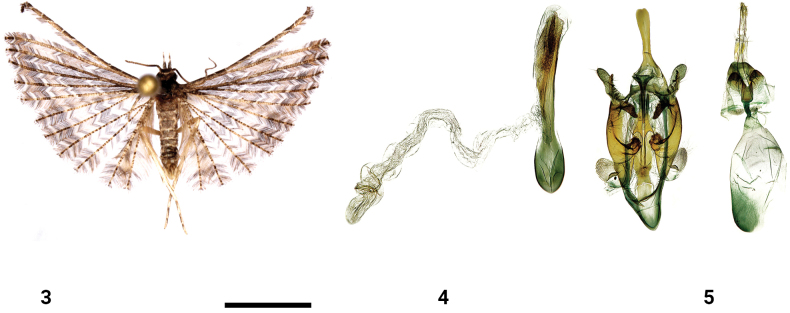
*Alucitasroczki* Ustjuzhanin & Kovtunovich, sp. nov. **3** adult male, holotype, NECJU**4** male genitalia, holotype, NEJCU, preparation slide no. 230702 **5** female genitalia, paratype, NEJCU, preparation slide no. 230703. Scale bar: 5 mm.

##### Differential diagnosis.

**Figures 6–8.**
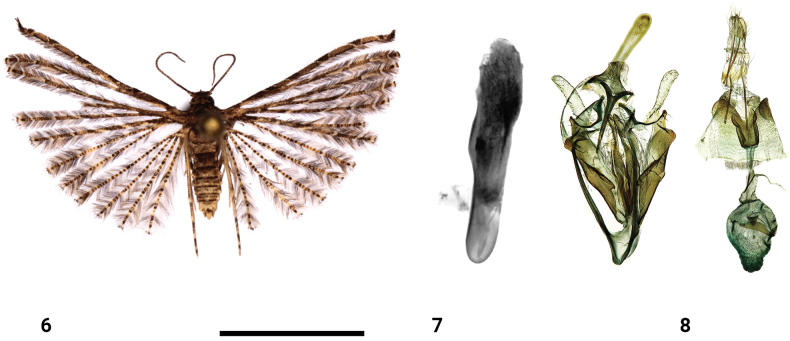
*Alucitamegaphimus* (Hering, 1917), stat. rev. **6** adult female **7** male genitalia **8** female genitalia. Scale bar: 5 mm.

*Alucitasroczki* shares a mottled greyish brown wing colouration with *Alucitaseychellensis* (T.B. Fletcher 1910) (illustrated in Ustjuzhanin and Kovtunovich 2016) and *Alucitamegaphimus* (Hering, 1917) (Figs 6–8). However, it can be distinguished from these species by the darkened terminal band present on all wings and by its larger size. The most reliable distinguishing feature lies in the male genital structure. While the general structure of the male genitalia is reminiscent of *A.seychellensis*, *A.sroczki* has narrower valves, a shorter gnathos, and a set of complex sacculus structures with serrated spiky forms, as well as a narrower aedeagus. In the female genitalia, the new species differs from *A.seychellensis* and *A.megaphimus* in the deep V-shaped notch on the outer edge of the antrum.

##### External characters.

The head, thorax, and tegulae are covered with clinging grey-brown hairs. Labial palpi are dark-grey and measure 2.5–3 × the longitudinal eye diameter. The third segment is thin, long, and belted basally and apically with narrow white scales. The antennae are dark brown. The wingspan ranges from 16 to 20 mm (holotype 18 mm). All wings exhibit a greyish brown colouration, with four and six distinct pale transverse zigzag bands on the fore wing and hind wing, respectively. The wings are noticeably darkened distally. The fringe on the lobes of all wings features alternating portions of pale yellow and dark brown hairs. The hind legs are pale yellow.

##### Male genitalia.

The uncus is simple, long, and medially narrowing, with a widened distal end that bears a small notch. The gnathos is slightly shorter than the uncus and narrow, tapering to an acute apex. The gnathos arms are short and wide. The valves are simple and short, measuring half the length of the uncus. The distal portion of the sacculus is expanded as a forked structure. The outer portion of this fork is narrower, internally serrated and terminating in an acute and slightly inwardly bent apex. The inner portion of the fork is wider and finger-like, also serrated on the inside. The basal portion of the sacculus is wide, with a globular sclerotised formation covered with tiny sharp needles. The anellus arms are long, equal in length to the gnathos, and wide at the base, gradually narrowing. The saccus is slightly elongated and caudally rounded. The aedeagus is straight, basally widened, and 1.5 × longer than the uncus. The cornutus is needle-like, distinctive, and occupies most of the aedeagus.

##### Female genitalia.

The papillae anales are narrow and elongated. The posterior apophyses are long and thin, approximately equal in length to the anterior apophyses. The antrum is tubulate and sclerotised, with a narrow V-shaped notch on the outer edge. The ductus is very short, slightly shorter than the antrum, and the ductus seminalis passes from the confluence of the ductus into the bursa. The bursa is oval and very large, featuring two narrow ribbon-like signa.

##### Distribution.

The species was found in Cameroon only.

##### Flight period.

The species was sampled in April and October.

##### Etymology.

The species name is a noun in apposition. It is named in honour of the curators from NECJU, Ewelina Sroka and Karolina Sroka, who crucially contributed to the processing of the abundant moth material collected on Mount Cameroon and several other Afrotropical localities. The name ’sroczki’ refers to the nickname commonly used for the twin sisters.

##### Note.

Previously, Ustjuzhanin and Kovtunovich (2017) erroneously synonymised *A.megaphimus* with *A.seychellensis*. Later, PU and VK re-examined their characters in more detail, and therefore we consider *Alucitamegaphimus* (Hering, 1917), stat. rev., as a separate species.

#### ﻿ Alucita fako

Taxon classificationAnimaliaLepidopteraAlucitidae

Ustjuzhanin & Kovtunovich sp. nov.

8AEAC9FB-B19B-5811-992D-FEBBBBC0A6CA

https://zoobank.org/EF4D5685-1041-4833-B6A4-965EABD0DF69

Figs 9–11

##### Type material.

***Holotype*** • ♂, (NECJU 230704), Cameroon, Ekonjo, 1150 m a.s.l., Mount Cameroon, 4.0881°N, 9.1168°E, 25.X.2017, lgt. V. Maicher, S. Delabye. ***Paratypes*** • 1 ♀, (NECJU 230705), Cameroon, Bamboo Camp, 350 m a.s.l., Mount Cameroon, 4.0879°N, 9.0505°E, 12 –20.XII.2014, lgt. V. Maicher, Sz. Sáfián, Š. Janeček, R. Tropek.

**Figures 9–11.**
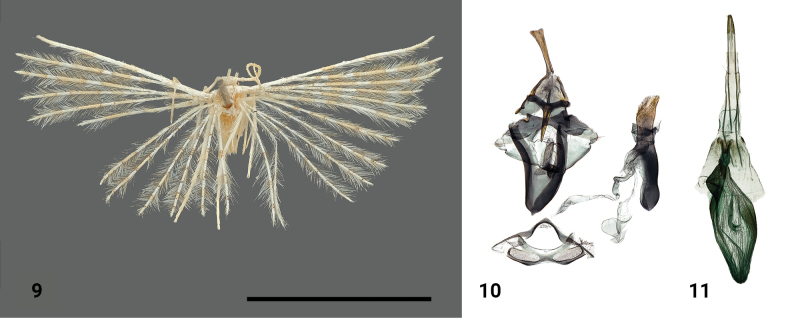
*Alucitafako* Ustjuzhanin & Kovtunovich, sp. nov. **9** adult male, holotype, NECJU**10** male genitalia and structures of the segment VIII of the male abdomen, holotype, NEJCU, preparation slide no. 230704 **11** female genitalia, paratype. Scale bar: 5 mm.

• 1 ♀, (NECJU), same data as holotype • 3 ♀, (NECJU, CUK), Cameroon, PlanteCam, 1100 m a.s.l., Mount Cameroon, 4.1175°N, 9.0709°E, 11–18.XII.2014, lgt. V. Maicher, Sz. Sáfián, Š. Janeček, R. Tropek • 4 ♀, (NECJU, CUK), Cameroon, Elephant Camp, 1850 m a.s.l., Mount Cameroon, 4.1170°N, 9.0729°E, 19–24.XI.2014, lgt. V. Maicher, Sz. Sáfián, Š. Janeček, R. Tropek • 1 ♀, (NECJU), Ekonjo, 1150 m a.s.l., Mount Cameroon, 4.0881°N, 9.1168°E, 24.X.2017, lgt. V. Maicher, S. Delabye • 1 ♀, (CUK), Crater Lake, 1500 m a.s.l., Mount Cameroon, 4.1443°N, 9.0717°E, 23–29.IV.2017, lgt. V. Maicher, S. Delabye.

##### Differential diagnosis.

In the male genitalia, the new species exhibits great similarity to *Alucitaescobari* Ustjuzhanin & Kovtunovich, 2018, from which it differs in the more reduced notch in the top of the uncus and in the caudally acute saccus. In contrast, in *A.escobari* the notch on the top of the uncus is clearly expressed, triangular, and the saccus is caudally smooth, oval, and not acute. Additionally, these two moth species are clearly distinct externally. In the female genitalia, the new species closely resembles *Alucitabesongi* Ustjuzhanin & Kovtunovich, 2018 and *Alucitajaneceki* Ustjuzhanin & Kovtunovich, 2018. From the former, it differs in the oval, elongated bursa copulatrix and the absence of small signa in it, while in *A.besongi* the bursa copulatrix is pear-like, its surface covered with tiny signa. From the latter, the new species differs in the shape of the bursa copulatrix and the antrum, in the new species the bursa copulatrix narrows caudally, while in *A.janeceki*, it has a rounded base. The antrum in the new species has a narrow V-shaped notch on the outer edge, while in *A.janeceki* the notch is wide. From both species, the new species differs in the very long posterior apophyses, in *A.besongi* and *A.janeceki* the anterior and posterior apophyses are equal in the length. Furthermore, the male genitalia of the new species are clearly different from those of *A.besongi* and *A.janeceki*.

##### External characters.

The head, thorax, and tegulae are white. Labial palpi are pale-yellow and measure twice the longitudinal eye diameter. The antennae are yellowish white. The wingspan ranges from 9 to 12 mm (holotype 11 mm). The wings are pale yellow, mottled, with alternating white and yellowish brown portions of scales. All lobes of the wings have small dark spots of scales on tips. The fringe on all lobes of the wings has alternating white and pale brown portions of hairs. The hind legs are white.

##### Male genitalia.

The uncus is long, distally extended, and apically with a poorly visible notch. The gnathos is slightly shorter than the uncus and apically acute. The gnathos arms are wide and slightly shorter than the gnathos itself. The valves are wing-like and apically have a bundle of thin needle-like setae. The anellus arms are wide, straight, and equal in length to the gnathos. The saccus is elongated and forms a narrow triangle, with an acute tip. The aedeagus is almost straight, obliquely cut apically, and without cornuti.

##### Female genitalia.

The papillae anales are narrow and elongated. The posterior apophyses are very long and thin. The antrum is sclerotised, with a narrow V-shaped notch on the outer edge. The ductus is very short, slightly shorter than the antrum, and the ductus seminalis extends distally inside the bursa copulatrix. The bursa copulatrix is oval, elongated, and noticeably narrows at the end, with numerous longitudinal long ribs inside.

##### Distribution.

The species was found in Cameroon.

##### Flight period.

The species was collected in May and from October to December.

##### Etymology.

The species is named after Fako, the local name of Mount Cameroon, which is the type locality of the species. The name aims to emphasise the importance of the area and encourage the protection of the species’ habitats.

#### ﻿ Alucita sedlaceki

Taxon classificationAnimaliaLepidopteraAlucitidae

Ustjuzhanin & Kovtunovich sp. nov.

6C3737D6-B8EE-51B1-BB5B-232DFD65D5FE

https://zoobank.org/9C428B4E-9960-4EC0-8C86-32BB3FB1DA6F

Figs 12 , 13

##### Type material.

***Holotype*** • ♂, (NECJU 230706), Cameroon, PlanteCam, 1100 m a.s.l., Mount Cameroon, 4.1175°N, 9.0709°E, 09–14.IV.2015. V. Maicher, Sz. Sáfián, Š. Janeček, R. Tropek. ***Paratypes*** • 1 ♂, (CUK), Cameroon, Mount Cameroon, Ekonjo, 1150 m a.s.l., 4.0881°N, 9.1168°E, 25.X.2017, lgt. V. Maicher, S. Delabye • 1 ♂, (CUK), Cameroon, Bamboo Camp, 350 m a.s.l., Mount Cameroon, 4.0879°N, 9.0505°E, 14–23.II.2016, lgt. V. Maicher, Sz. Sáfián, R. Tropek.

**Figures 12, 13.**
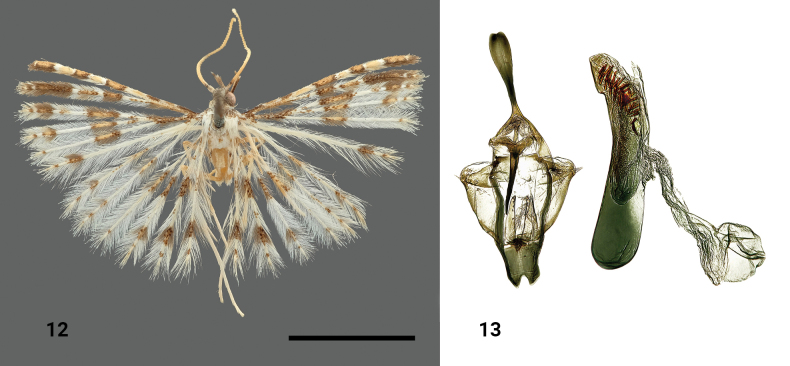
*Alucitasedlaceki* Ustjuzhanin & Kovtunovich, sp. nov. **12** adult male, holotype, NECJU**13** male genitalia, holotype, NEJCU, preparation slide no. 230706. Scale bar: 5 mm.

##### Differential diagnosis.

In terms of wing colouration, the species somewhat resembles *Alucitamischenini* Ustjuzhanin & Kovtunovich, 2018, but it differs in the length of the apical dark-brown portion of scales on the first and second lobes of the fore wing. In the new species, the portion on the second lobe is twice as long as that on the first lobe, while in *A.mischenini* it is equal to or even shorter than that on the first lobe. In the male genitalia, there is also a similarity to *A.mischenini*, but in the new species, the saccus has a distinct triangular notch caudally, and the aedeagus has an ordered arrangement of needle-like cornuti distally. In contrast, in *A.mischenini* the notch is absent on the saccus, and the cornuti in the aedeagus are tiny and chaotically disorganised.

##### External characters.

The head and thorax are brown, and the tegulae are white. The labial palpi are brown and measure 2.5 × longer than the longitudinal eye diameter. The third segment is short, isolated, and directed upwards. The antennae are yellowish brown and serrated. The wings are white, with black and brown portions of scales. The medial band is well-developed. The wingspan ranges from 12 to 16 mm (holotype 16 mm). The first lobe on the fore wing has alternating brown and yellow rectangular portions. The apical dark brown portion on the first lobe is half as long as the apical portion on the second lobe. The fore wings are basally darkened with dark-brown scales. Medially, they have a wide brown band, which is missing on the sixth lobe. On the hind wings, this band is positioned closer to the base of the wing. All wings have a dark brown subterminal band that is broken in the fifth lobe of the forewing and the third lobe of the hindwing, with the small dark spots of scales subapically on all lobes of all wings. The fringe on the wings is pale, with only the banded portions being brown. The hind legs are yellowish white.

##### Male genitalia.

The uncus is long, basally and medially narrow, and distally widened, with a small notch at the apex. The gnathos is narrow, apically acute, and equal in length to the uncus. The gnathos arms are short and wide. The valves are short, wide, and wing-like. The anellus arms are long, slightly shorter than the gnathos, but significantly wider than it, being basally wide and apically narrowing. The saccus is equal in length to the anellus arms, with a clearly expressed triangular notch caudally. The aedeagus is slightly concave medially and almost equal in length to the entire genital structure (excluding the uncus). The aedeagus large needle-like cornuti distally arranged in an orderly array.

##### Distribution.

The species was found in Cameroon only.

##### Flight period.

The species was collected in February, April, and October.

##### Etymology.

The species is named in honour to Ondřej Sedláček, a recognised ornithologist and entomologist with experience from many African countries. On Mount Cameroon, he established several ongoing ecological research projects and was instrumental in helping local communities to understand how to protect the unique local ecosystems in which they live.

#### ﻿ Alucita tonda

Taxon classificationAnimaliaLepidopteraAlucitidae

Ustjuzhanin & Kovtunovich sp. nov.

8E6F9F66-25BA-5196-B7C2-57D14EDD72E0

https://zoobank.org/B1D0EEEE-1911-44F8-85E5-9A611A80A993

Figs 14 , 15

##### Type material.

***Holotype*** • ♀, (NECJU 230707), Cameroon, Drink Gari, 650 m a.s.l., Mount Cameroon, 4.1014°N, 9.0610°E, 06–15.II.2016, lgt. V. Maicher, Sz. Sáfián, Š. Janeček, R. Tropek.

**Figures 14, 15.**
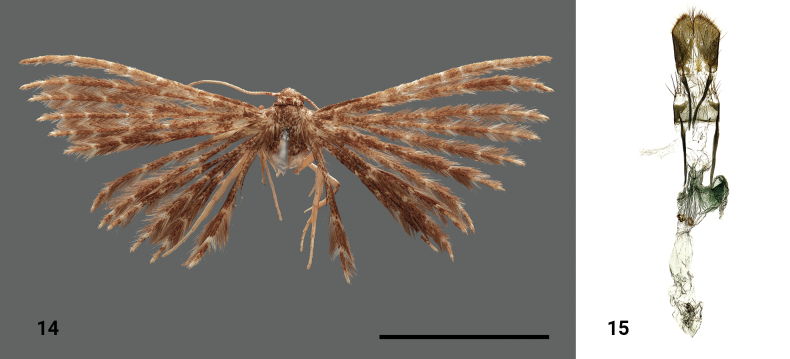
*Alucitatonda* Ustjuzhanin & Kovtunovich, sp. nov. **14** adult male, holotype, NECJU**15** male genitalia, holotype, NEJCU, preparation slide no. 230707. Scale bar: 5 mm.

##### Differential diagnosis.

The dark colour of the wings in this species shows some similarity to *Alucitaacalyptra* Meyrick, 1913, but the new species lacks the zigzag bands on the wings’ distal parts. In terms of the female genitalia, the new species stands out with its unusual asymmetric structure of the antrum, which has no analogues among known species.

##### External characters.

The head, thorax, and tegulae are dark brown. The labial palpi are short, straight, and slightly longer than the longitudinal eye diameter. The antennae are brown. The wingspan is 16 mm, and the wings are dark brown. Narrow pale longitudinal bands are present on the lobes of all wings. The fringe on the lobes of all wings is greyish brown. The hind legs are pale yellow.

##### Female genitalia.

The papillae anales are wide. The posterior apophyses are short, thick, and slightly shorter than the anterior apophyses. The antrum is asymmetric, sclerotised, and distally tubulate, with a small triangular notch in the middle. The medium portion of the antrum is very wide, with the right half distinctively protruding to the side, creating a structural asymmetry. The lower portion of the antrum is membranous and bears two round sclerotised plaques. The ductus is short, almost invisible, and passes into a narrow membranous bursa copulatrix, with no signa observed.

##### Distribution.

The species was found in Cameroon only.

##### Flight period.

The species was collected in February.

##### Etymology.

The species name is a noun in apposition, given in honour of Antonín “Tonda” Tropek, who is RT’s father.

#### ﻿ Alucita erzayi

Taxon classificationAnimaliaLepidopteraAlucitidae

Ustjuzhanin & Kovtunovich sp. nov.

B693BB21-D85A-5D8B-B43F-0449D542D9DA

https://zoobank.org/4527A604-C418-43BD-9CFF-9EF96A06A7C3

Figs 16 , 17

##### Type material.

***Holotype*** • ♂, (NECJU 230708), Cameroon, PlanteCam, 1100 m a.s.l., Mount Cameroon, 4.1175°N, 9.0709°E, 11–23.IV. 2014, lgt. V. Maicher, Sz. Sáfián, Š. Janeček, R. Tropek.

**Figures 16, 17.**
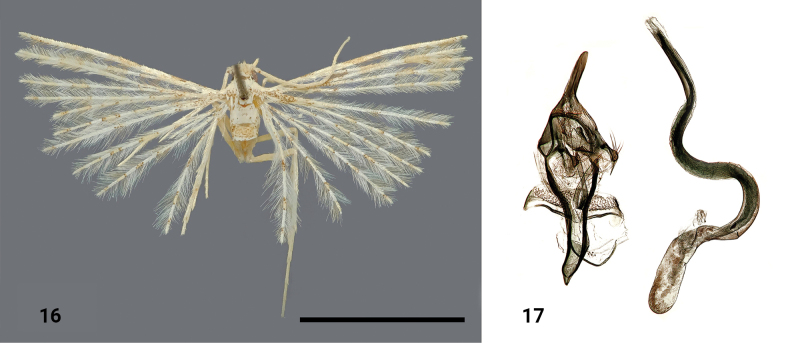
*Alucitaerzayi* Ustjuzhanin & Kovtunovich, sp. nov. **16** adult male, holotype, NECJU**17** male genitalia and structures of the segment VIII of the male abdomen, holotype, NEJCU, preparation slide no. 230708. Scale bar: 5 mm.

##### Differential diagnosis.

The male genital structures of this new species resemble those of *Alucitalongipenis* Ustjuzhanin & Kovtunovich, 2018. As in *A.longipenis*, the aedeagus of the new species is very long, but it is slightly shorter, has fewer curves, and is straight for the distal portion. Additionally, the saccus of *A.erzayi* is gently curved distally and apically acute, while in *A.longipenis* the saccus is bent and apically acute. The wingspan of the new species is 14 mm, whereas it is 18–23 mm for *A.longipenis*.

##### External characters.

The head, thorax, and tegulae are yellowish white. The labial palpi are thin, straight, and twice as long as the longitudinal eye diameter. The antennae are pale yellow. The wingspan is 14 mm, and the wings are pale yellow, interspersed with brown strokes and spots. Two small dark brown patches are present in the basal portion of the costa of the first lobe of the fore wing. Indistinct pale brown regions of scales are present in the medial and distal portions of the first two lobes. The fringe on the wings is pale yellow, and the hind legs are pale yellow.

##### Male genitalia.

The uncus is relatively long and evenly wide throughout its length, with a rounded apex. The gnathos is long and narrow. The valves are reduced. The anellus arms are long and evenly narrow throughout their length. The saccus is long, elongated, and smoothly bent caudally, with a clearly acute apex. The aedeagus is very long, ~ 4 × longer than the entire genital structure, forming two arched curves in the medium part. No cornuti are present.

##### Distribution.

The species was found in Cameroon only.

##### Flight period.

The species was collected in April.

##### Etymology.

The species is a noun in apposition. ‘Erzayi’ is a word in the Bakweri language, which is the dominant local language in the Mount Cameroon region, and it translates to “feather”. This corresponds with the appearance of many-plumed moths’ feather-like characteristic wing lobes.

#### ﻿ Alucita sokolovi

Taxon classificationAnimaliaLepidopteraAlucitidae

Ustjuzhanin & Kovtunovich sp. nov.

4A31C78D-2166-57FE-A603-31691E12B2E0

https://zoobank.org/3D9F4858-D646-4A23-9620-E3C883642BD0

Figs 18 , 19

##### Type material.

***Holotype*** • ♂, (NECJU 230709), Cameroon, Mann’s Spring, 2200 m a.s.l., Mount Cameroon, 4.1428°N, 9.1225°E, 16–21. IV. 2017, lgt. V. Maicher, P. Potocký, S. Delabye. ***Paratypes*** • 2 ♂, (NECJU, CUK), Cameroon, Crater Lake, 1500 m a.s.l., Mount Cameroon, 4.1443°N, 9.0717°E, 17–25. II. 2017, lgt. P. Potocký, Sz. Sáfián, R. Tropek, J. Mertens, Š. Janeček • 1 ♂, (CUK), Cameroon, Mapanja, 1850 m a.s.l., Mount Cameroon, 4.1157°N, 9.1315°E, 13.V.2017, lgt. V. Maicher, P. Potocký, S. Delabye.

**Figures 18, 19.**
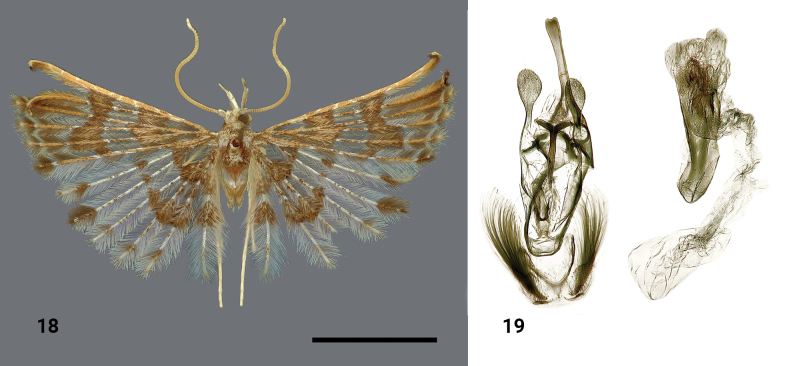
*Alucitasokolovi* Ustjuzhanin & Kovtunovich, sp. nov. **18** adult male, holotype, NECJU**19** male genitalia and structures of the segment VIII of the male abdomen, holotype, NEJCU, preparation slide no. 230709. Scale bar: 5 mm.

##### Differential diagnosis.

The wing colour of this species slightly resembles *Alucitajana* Ustjuzhanin & Kovtunovich, 2020, but it can be distinguished by the widened distal band on the fore wing, whereas in *A.jana* it narrows at the fourth-fifth lobe. Additionally, *A.sokolovi* has a band in the medial portion of the wing, which is absent in *A.jana*. The labial palpi of the new species are 3 × longer than the longitudinal eye diameter, compared to only 1.5 × in *A.jana*. In the male genitalia, the structure of the aedeagus and the shape of the gnathos of *A.sokolovi* slightly resemble *Alucitabokwango* Ustjuzhanin & Kovtunovich, 2020. However, *A.sokolovi* can be differentiated by its uncus, which is widened on the top, and the wide oval apical portions of the valves, while in *A.bokwango* the uncus is narrow throughout its length, and the valves’ apices are less widened.

##### External characters.

The head has white shiny scales. The thorax and tegulae are covered with pale brown clinging scales. The labial palpi are wide, long, ~ 3 × as long as the longitudinal eye diameter, white inside and brown outside. The third segment is isolated and apically acute. The antennae are yellowish brown, and the scape is wide and flattened. The wingspan is 17–18 mm (holotype 17 mm), and the wings are pale brown. There are two clearly expressed wide brown bands on the fore wings, distally and basally. The fore wings are apically framed with a white subapical zigzag. The hind wings are noticeably paler than the fore wings, with a brown band widening towards the last three lobes. There are bundles of brown hairs on the lobes, both distally and basally, with white fringes between them. The hind legs are pale yellow.

##### Male genitalia.

The uncus is long, noticeably exceeding the gnathos in length, and is distally slightly widened with a small apical notch. The gnathos is narrow and apically acute. The valves are long and membranous, apically smoothly forming a wide oval shape. The anellus arms are thin and straight. The saccus is caudally oval. The aedeagus is short, almost straight, and has two spiky cornuti.

##### Distribution.

The species was found in Cameroon only.

##### Flight period.

The species was collected in February, April, and May.

##### Etymology.

The new species is named after Vasily Igorevich Sokolov (Moscow, Russia), the famous Russian ichthyologist and bioresource recovery specialist.

#### ﻿ Alucita hirsuta

Taxon classificationAnimaliaLepidopteraAlucitidae

Ustjuzhanin & Kovtunovich sp. nov.

0208749E-AFC2-51AA-8E8F-2FB162999AE7

https://zoobank.org/0B0E031A-7DE7-49CE-8CA7-A36DEF703D7D

Figs 20 , 21

##### Type material.

***Holotype*** • ♀, (NECJU 230710), Cameroon, Mapanja, 1850 m a.s.l., Mount Cameroon, 4.1157°N, 9.1315°E, 23.X.2017, lgt. V. Maicher, S. Delabye.

**Figures 20, 21.**
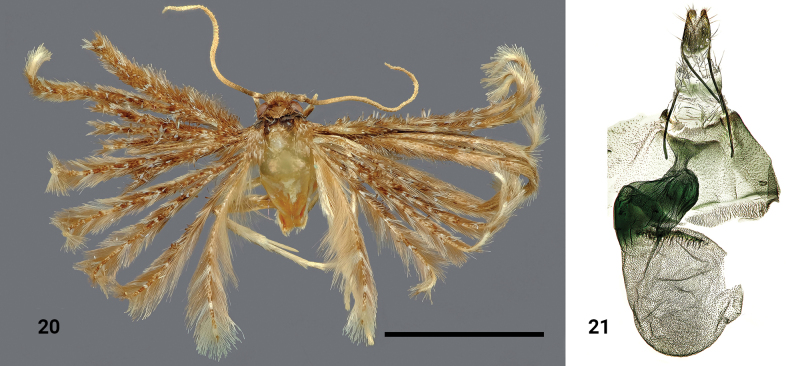
*Alucitahirsuta* Ustjuzhanin & Kovtunovich, sp. nov. **20** adult female, holotype, NECJU**21** female genitalia, holotype, NEJCU, preparation slide no. 230710. Scale bar: 5 mm.

##### Differential diagnosis.

The mushroom-like antrum and elongated crest-like signum of this species bear similarity to *Alucitaectomesa* (Hering, 1917) (illustrated in Ustjuzhanin and Kovtunovich 2016), but it can be distinguished by the wider ductus, the round bursa copulatrix, and numerous tiny spiky signa present in it. Additionally, the unique colouration of the wings sets *A.hirsuta* apart from all other African Alucitidae species.

##### External characters.

The head, thorax, and tegulae are dark brown. The labial palpi are short, slightly longer than the longitudinal eye diameter. The antennae are brown. The wingspan is 14 mm, and the wings have a reddish brown appearance. The lobes of all wings bear protruding tousled dark-brown hairs, especially dense on the first two lobes of the fore wings, creating the appearance of a shaggy moth. There are narrow, poorly visible pale longitudinal bands on all wings. The fringe on all wings ranges from pale to dark brown. The hind legs are yellow.

##### Female genitalia.

The papillae anales are narrowly triangular in shape. Both the posterior and anterior apophyses are of equal length, thick, and straight. The antrum is wide and mushroom-like. The ductus is wide, corrugated, and strewn with narrow strands. The ductus seminalis passes from the middle of the ductus. The bursa copulatrix is round, with a robust crest-like signum located in the upper part of the bursa, near the confluence of the ductus. Numerous tiny spiky signa densely cover the entire surface of the bursa.

##### Distribution.

The species was found in Cameroon only.

##### Flight period.

The species was collected in October.

##### Etymology.

The species name is derived from Latin ’hirsute’ (shaggy, bristly, hairy). It refers to the appearance of the adult moth, with tousled protruding dark-brown hairs on the wings, reminiscent of a hairy and shaggy moth.

#### ﻿ Alucita potockyi

Taxon classificationAnimaliaLepidopteraAlucitidae

Ustjuzhanin & Kovtunovich sp. nov.

5D7E07D1-3B58-55E7-A6F1-210104270F90

https://zoobank.org/F8D2F968-7B4B-4ED1-9861-105B9BBF9972

Figs 22 , 23

##### Type material.

***Holotype*** • ♂, (NECJU 230711), Cameroon, Mexico Camp, 30 m a.s.l., Bimbia-Bonadikombo, 3.9818°N, 9.2625°E, 10.X.2017, lgt. V. Maicher, S. Delabye.

**Figures 22, 23.**
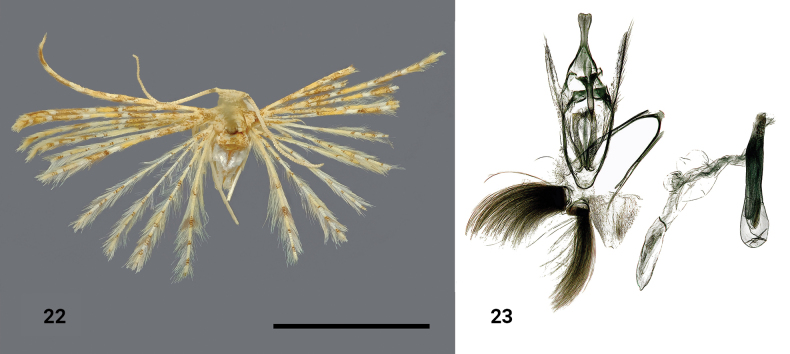
*Alucitapotockyi* Ustjuzhanin & Kovtunovich, sp. nov. **22** adult male, holotype, NECJU**23** male genitalia and structures of the segment VIII of the male abdomen, holotype, NEJCU, preparation slide no. 230711. Scale bar: 5 mm.

##### Differential diagnosis.

The yellow wing colour of this species resembles *Alucitacompsoxantha* Meyrick, 1924, but it can be distinguished by the differences in the position of the bands. In the male genitalia, *A.potockyi* shares similarities with *Alucitatesserata* (Meyrick, 1918) in the short uncus widening towards the apex and in the saccus shape, but it clearly differs in the apically tapered gnathos, long narrow valves and the long aedeagus that exceeds the genital structure in its length. In comparison, *A.tesserata* has a gnathos that strongly widens apically, short and wide valves, and a noticeably smaller aedeagus relative to the genital structure (see Ustjuzhanin et al. 2020a for the genitalia illustration).

##### External characters.

The forehead is covered with white clinging scales, while the nape bears protruding yellow-brown hairs. The thorax and tegulae are yellowish brown. The labial palpi are short, ~ 1.5 × as long as the longitudinal eye diameter, with the third segment acute and framed with brown scales. The antennae are yellow, with only the basal area above the scape adorned with dark-brown scales. The wingspan measures 14 mm, and the wings have a yellowish brown colouration. There is a narrow white band medially on the fore wings (potentially even more bands can be present, but it is difficult to distinguish them clearly on the single available specimen of a mediocre quality), and alternating brown and yellowish portions of scales are present in the distal half of the fore wings. The wings’ basal areas are covered with brown scales. The hind wings are slightly paler than the fore wings, with alternating brown and yellow portions of scales along all lobes. The fringe on all wings is yellow, and the hind wings appear pale-yellow.

##### Male genitalia.

The uncus is short, widening apically. The gnathos is robust, sharply narrowing apically. The valves are narrow, long, and poorly sclerotised. The gnathos arms are short and narrow-triangular. The anellus arms are thin, long, straight, and apically form axe-shaped extensions. The saccus is caudally oval. The aedeagus is long, slightly longer than the genital structure, and bears a series of tiny transverse spiky cornuti distally, along with two big needle-like cornuti positioned along the aedeagus medially.

##### Distribution.

The species was found in Cameroon only.

##### Flight period.

The species was collected in October.

##### Etymology.

The species is named after the Czech lepidopterist Pavel Potocký Sr., in appreciation of his long-term support with moth preparation and identification in various projects of RT’s research group.

### ﻿Other species newly recorded on Mount Cameroon

#### ﻿ Alucita agassizi

Taxon classificationAnimaliaLepidopteraAlucitidae

Ustjuzhanin & Kovtunovich, 2018

982769B3-5891-58FD-9D99-1B717EE0C855

Alucita agassizi Ustjuzhanin & Kovtunovich, 2018: 169. Type locality: Tanga, E Usambara, Tanzania.

##### Type material examined.

***Holotype*** • ♀, Natural History Museum of United Kingdom, London, UK (NHMUK), examined by the authors (illustrated in Ustjuzhanin et al. 2018b).

##### Other material examined.

1 ♂ (CUK), 1 ♀ (NECJU), Cameroon, Bamboo Camp, 350 m a.s.l., Mount Cameroon, 4.0879°N, 9.0505°E, 19.XII.2014 • 2 ♂ (NECJU), 12–20.XII.2014; 2 ♂ (CUK), 17–23.IV.2015, lgt. V. Maicher, Sz. Sáfián, S. Janeček, R. Tropek • 1 ♀ (CUK), Cameroon, PlanteCam Camp, 1100 m a.s.l., Mount Cameroon, 4.1175°N, 9.0709°E, 09–14.IV.2015, lgt. V. Maicher, Sz. Sáfián, S. Janeček, R. Tropek.

##### Distribution.

The species was found in Tanzania and Cameroon.

##### Note.

New species for Cameroon.

#### ﻿ Alucita dohertyi

Taxon classificationAnimaliaLepidopteraAlucitidae

(Walsingham, 1909)

D4C6DEDB-ECA1-5FD7-BA4A-71C09E840ACE

Orneodes dohertyi Walsingham, 1909: 174. Type locality: Ibea, Kikuyu, Escarpment, E Africa, [Kenya].
Orneodes decaryella Viette, 1956: 89. Type locality: Madagascar.

##### Type material examined.

***Holotype*** • ♂, NHMUK, examined by the authors.

##### Other material examined.

5 ex. (NECJU, CUK), Cameroon, Mount Cameroon, Mapanja, 1850 m a.s.l., 4.1157°N, 9.1315°E, 28.X.2017, lgt. V. Maicher, S. Delabye.

##### Distribution.

The species is known from Tanzania, Uganda, Kenya, Madagascar, Republic of South Africa (De Prins and De Prins 2023), and Cameroon.

##### Note.

New species for Cameroon.

#### ﻿ Alucita plumigera

Taxon classificationAnimaliaLepidopteraAlucitidae

(Strand, 1913)

50882104-9A4E-5864-A3C3-8A0403A0D128

Orneodes plumigera Strand, 1913: 63. Type locality: Alén, Equatorial Guinea.

##### Type material examined.

***Holotype*** • ♂, Museum für Naturkunde, Berlin, Germany (MfN), examined by the authors.

##### Other material examined.

(NECJU, CUK) 2 ♂, Cameroon, PlanteCam Camp, 1100 m a.s.l., Mount Cameroon, 4.1175°N, 9.0709°E, 11–18.XII.2014, lgt. V. Maicher, Sz. Sáfián, Š. Janeček, R. Tropek • 6 ex., Cameroon, Bamboo Camp, 350 m a.s.l., Mount Cameroon, 4.0879°N, 9.0505°E, 17–23.IV.2015, lgt. V. Maicher, Sz. Sáfián, Š. Janeček, R. Tropek • 2 ex., Cameroon, Mount Cameroon, Bamboo Camp, 350 m a.s.l., 29.I.–07. II.2016, lgt. Sz. Sáfián, R. Tropek, V. Maicher • 1 ♂, Cameroon, PlanteCam Camp, 1100 m a.s.l., Mount Cameroon, 4.1175°N, 9.0709°E, 09–14.IV.2015, lgt. V. Maicher, Sz.Sáfián, S. Janeček, R. Tropek • 5 ex., Cameroon, Bamboo Camp, 350 m a.s.l., Mount Cameroon, 4.0879°N, 9.0505°E, 12–20.XII.2014, lgt. V. Maicher, Sz. Sáfián, S. Janeček, R. Tropek • 1 ♂, 2 ♀s, Cameroon, Mount Cameroon, Mapanja, 1850 m a.s.l., 4.1157°N, 9.1315°E, 23–28.X.2017, lgt. V. Maicher, S. Delabye • 1 ♀, Cameroon, Mount Cameroon, Ekonjo, 1150 m a.s.l., 4.0881°N, 9.1168°E, 21.X.2017, lgt. V. Maicher, S. Delabye • 1 ♀, Cameroon, Mount Cameroon, Drink Gari, 650 m a.s.l., 4.1014°N, 9.0610°E, 06–15.II.2016, lgt. Sz. Sáfián, R. Tropek, V. Maicher.

**Figures 24, 25.**
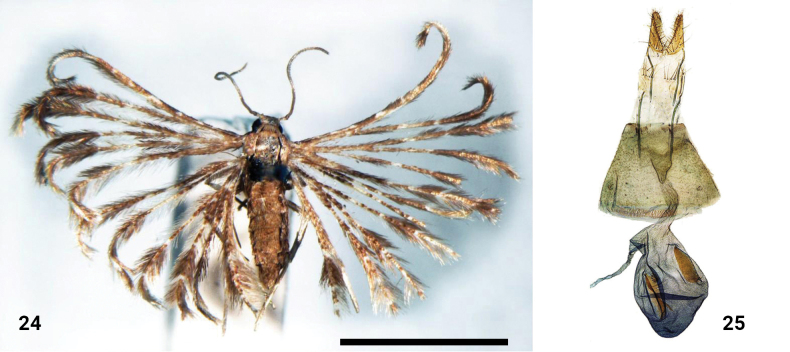
*Alucitamolliflua* (Meyrick, 1927) **24** adult female, holotype **25** female genitalia, holotype. Scale bar: 5 mm.

##### Distribution.

The species occurs in Equatorial Guinea and Cameroon.

##### Note.

New species for Cameroon.

#### ﻿ Alucita rhaptica

Taxon classificationAnimaliaLepidopteraAlucitidae

(Meyrick, 1920)

22DBCC3C-B28A-52C4-9AE5-056B962D7D35

Orneodes rhaptica Meyrick, 1920: 82. Type locality: Kwale County, [Kenya].

##### Type material examined.

***Holotype*** • ♀, Museum National d′Histoire Naturelle, Paris, France (MNHN), examined by the authors.

##### Other material examined.

1 ♀ (NECJU), Cameroon, Bamboo Camp, 350 m a.s.l., Mount Cameroon, 4.0879°N, 9.0505°E, 17–23.IV.2015, lgt. V. Maicher, Sz. Sáfián, S. Janeček, R. Tropek. • 1 ♀, 20.XII.2014, same data as the previous specimen.

##### Distribution.

The species occurs in Tanzania, Malawi, Republic of South Africa (De Prins and De Prins 2023), and Cameroon.

##### Note.

New species for Cameroon. Female genitalia of this species were illustrated in Ustjuzhanin et al. (2020a).

### ﻿Key to identify *Alucita* species in the Mount Cameroon area (males only)

In addition to the 36 species of *Alucita* previously reported from the Mount Cameroon area in Ustjuzhanin et al. (2018a, 2020a), Ustjuzhanin and Kovtunovich (2016), and in this study, the identification key also includes the only other species known from Cameroon, *Alucitailluminatrix* (Meyrick, 1929) described from Bitje, South Region, Cameroon (DePrins and DePrins 2023). Although this species has not been recorded in the Mount Cameroon area, we have included it in the identification key. On the other hand, *Alucitatatjana* Ustjuzhanin & Kovtunovich, 2020, *A.pyrczi*, *A.tonda*, and *A.hirsuta* are not included in the identification key despite their occurrence on Mount Cameroon, because only females are known for these species.

**Table:** 

1	Wingspan ≥ 20 mm	**2**
–	Wingspan < 20 mm	**5**
2	Wingspan ≥ 30 mm	**3**
–	Wingspan < 30 mm	**4**
3	Hind wings basally whitish with blackish grey bars distally. A postmedian transverse narrow brown band in hind wings. Hind wings distally framed in a wide dark brown band. In female genitalia, antrum oval, large, wide, almost equal to bursa copulatrix; ductus narrow, short	***A.illuminatrix***
–	Hind wings basally brown. Several transverse lightened bands running down in the middle of hind wings. Hind wings distally framed by a narrow brown band. In female genitalia, antrum cup-like; ductus wide, short	***A.dohertyi***
4	Wings predominantly yellowish orange	***A.coffeina***
–	Wings without yellow or orange colouration	**5**
5	Wings mostly dark brown and black. Wings dark, brown. In male genitalia, aedeagus straight, shorter than the entire genital apparatus	***A.bokwango***
–	Wings pale, almost white. In male genitalia, aedeagus strongly curved, 5 × longer than the entire genital apparatus	***A.longipenis***
6	Male genitalia with reduced valves	**7**
–	Male genitalia with developed valves	**11**
7	Aedeagus 4 × longer than the entire genital structure, with 2 arched curves in its medium part. Wingspan 14 mm	***A.erzayi***
–	Aedeagus equal, slightly longer or slightly shorter than the entire genital structure, straight or slightly curved	**8**
8	Saccus long, elongated, caudally acute	**9**
–	Saccus short, caudally rounded	**10**
9	Uncus finger-like, of equal width throughout its length. Aedeagus with 2 small horn-like cornuti. Anellus arms short, narrow, arched, apically acute. Wings mottled brown and white. Wingspan 12 mm	***A.zuza***
–	Uncus distally extended, apically with a small notch in the middle. Aedeagus with 1 long needle-like cornutus, exceeding the length of aedeagus. Anellus arms very short, with wide lobes, equally in length to gnathos. Wings yellowish brown, with 3 transverse white bands. Wingspan 12–13 mm	***A.fokami***
10	Saccus with a small notch caudally. Aedeagus with long needle-like cornuti. Gnathos short, wide. Well expressed white belts on abdominal tergites. Wings dark brown with portions of white scales. Wingspan 13–15 mm	***A.deja***
–	Saccus caudally without a notch. Aedeagus with a cluster of tiny cornuti. Gnathos not expressed. Abdomen without any white belts. Wings greyish white, basally darkened with brown scales. Wingspan 10–12 mm	***A.janeceki***
11	Uncus apically expanded	**12**
–	Uncus equally wide throughout its length, apex not expanded	**25**
12	Valves wing-like, wide	**13**
–	Valves not wing-like: finger-like, narrow or wide, short or long	**19**
13	Saccus short	**14**
–	Saccus noticeably elongated	**15**
14	Valves apically with long needle-like setae. Uncus long, paddle-like, apically with a smooth edge. Aedeagus relatively short, almost straight, distally with a cluster of tiny needle-like cornuti. Wing pattern uniquely greyish white with pale grey-brown basal regions, wide brown medial bands, and distal portion of forewings outlined in dark brown. Wingspan 16–18 mm	***A.ludmila***
–	Valves apically without needle-like setae. Uncus long, basally narrow, distally extended, apically with a weak notch. Saccus wide, caudally with a small notch. Aedeagus short, slightly bent in the middle, distally with a cluster of tiny spiky cornuti. Wings mottled, with a clearly expressed medial band, hind wings basally lightened. Wingspan 12–15 mm	***A.mischenini***
15	Saccus not solid, caudally not closed, discontinuous. Anellus arms long, slightly bent inwards. Aedeagus distally and apically with tiny needle-like cornuti. Wings white, with wide dark brown bands. Wingspan 18 mm	***A.zinovievi***
–	Saccus solid, caudally closed, not discontinuous	**16**
16	Saccus with a triangle notch caudally. Anellus arms slightly shorter than gnathos. Aedeagus distally with orderly arranged big needle-like cornuti. Wings white, with black and brown portions of scales, a medial band well developed. Wingspan 12–16 mm	***A.sedlaceki***
–	Saccus without a notch caudally	**17**
17	Saccus distinctly acute caudally. Gnathos slightly shorter than uncus, apically acute, with wide arms. Anellus arms wide, straight, equal to gnathos in its length. Aedeagus almost straight, obliquely cut apically, without cornuti. Wings pale yellow, mottled, with alternating white and yellowish brown portions of scales. Wingspan 9–12 mm	***A.fako***
–	Saccus caudally rounded, not acute	**18**
18	Gnathos wide, slightly shorter than uncus. Aedeagus apically without protruding spikes. Anellus arms relatively wide, slightly shorter than the gnathos, slightly bent inwards, apically narrowing. Wings yellowish brown, medially with a clearly expressed transverse brown arched band on all wings. Wingspan 14–16 mm	***A.escobari***
–	Gnathos narrow, equally long as uncus. Aedeagus apically with sharp thin spikes protruding outwards. Anellus arms wide, short. Wings yellowish brown, with 4 transverse white bands.Wingspan 18 mm	***A.bakweri***
19	Valves wide, short, not extending beyond base of uncus	**20**
–	Valves narrow, long, extending beyond base of uncus	**22**
20	Saccus elongated, gnathos narrow. Sacculus of complicated structure, anellus arms long, distally narrowing. Cornutus needle-like, occupying most of aedeagus. Wings elongated, greyish brown, mottled, with 4 clearly expressed transverse zigzag pale bands on all wings. Wingspan 16–20 mm	***A.sroczki***
–	Saccus not elongated, caudally rounded, gnathos wide	**21**
21	Anellus arms wide, gnathos arms short. Aedeagus with a cluster of tiny needle-like cornuti medially. Uncus apically with a small notch. Wingspan 16–20 mm	***A.plumigera***
–	Anellus arms narrow, gnathos arms long, arched. Aedeagus with a cluster of needle-like cornuti distally. Uncus with 2 small notches apically. Wingspan 15 mm	***A.jana***
22	Uncus long, noticeably exceeding length of gnathos. Valves smoothly forming extended ovals apically. Aedeagus short, almost straight, with 2 spiky cornuti. Anellus arms thin, straight. Saccus caudally oval. Wings pale brown. Fore wings apically framed in a white zigzag rim, basally and distally with 2 distinct wide brown bands on fore wings. Hind wings noticeably paler than fore wings, medially with a brown band extending to the last 3 lobes. Wingspan 17–18 mm	***A.sokolovi***
–	Uncus short, equal to gnathos in length. Valves not extended apically	**23**
23	Uncus with a small notch apically. Gnathos arms short, shaped as narrow triangles. Anellus arms thin, long, apically forming hatchet-like extensions. Valves narrow, weakly sclerotised. Wings yellowish brown, medially with a narrow white band and alternating portions of brown and yellowish scales on fore wings. Hind wings slightly paler than fore wings. Wingspan 14 mm	***A.potockyi***
–	Uncus with two uncinate processes apically. Gnathos arms long, narrow, tapered to apices	**24**
24	Gnathos wide, slightly narrowing distally. Valves slightly narrowing apically. Aedeagus narrow, elongated, longer than the entire genital structure, with 1 distinct long cornutus and a cluster of tiny needle-like cornuti distally. Wings mottled, yellowish brown. First lobe of fore wings with clearly expressed elongated orange spots alternating with elongated dark brown spots and separated by white bands. Wingspan 10–11 mm	***A.olga***
–	Gnathos narrow, apically strongly extended. Apices of valves rounded. Aedeagus narrow, shorter the entire genital structure. Wings mottled, grey, with alternating dark and pale portions and a clearly expressed dark medial band. All wing lobes apically ending with a small dark brown spot. Wingspan 12–15 mm	***A.spicifera***
25	Saccus elongated	**26**
–	Saccus not elongated	**28**
26	Valves apically clearly extended, rounded. Gnathos narrow, apically acute. Anellus arms narrow, straight. Aedeagus 1.5 × shorter than the entire genital structure, without cornuti. Wings pale brown, with a clearly expressed medial white band. Wingspan 13–16 mm	***A.chloracta***
–	Valves simple, apically not extended or only slightly extended	**27**
27	Aedeagus long, almost 2 × as long as the entire genital structure. Uncus long, distally extended, apically slightly acute. Gnathos narrow, relatively long, apically acute. Valves wider at base. Anellus arms straight, long. Saccus elongated, oval. Wings pale brown, with white crossing lines and conspicuous elongated white patches separated by pale brown portions on the first lobe. Wingspan 8–10 mm	***A.besongi***
–	Aedeagus short, 1.5 × shorter than the entire genital structure, with a distinct large spiky cornutus in its middle part. Valves narrow, membranous, slightly extended apically. Gnathos narrow, equal to uncus in its length. Anellus arms long, straight. Wings elongated, mottled, dark grey, with clearly expressed pale transverse zigzag bands. Wingspan 13–14 mm	***A.megaphimus***
28	Saccus caudally saddle-shaped	**29**
–	Saccus caudally rounded	**30**
29	Uncus apically acute. Aedeagus short, wide, with a cluster of tiny cornuti distally. Valves narrow, longer than gnathos. Wings mottled, dark grey to almost black, distally with pale thin transverse zigzag bands. Wingspan 12–15 mm	***A.acalyptra***
–	Uncus apically rounded bluntly. Aedeagus long, narrow, without cornuti. Valves short, wide, equally long as gnathos. Wings mottled, dark grey, with portions of whitish scales, with a pale zigzag band along an outer edge of all wings. Each lobe apically ending with a small spot of dark scales. Wingspan 10–12 mm	***A.agassizi***
30	Valves apically narrow. Anellus arms undulated, long, reaching the centre of gnathos. Gnathos apically acute. Wings mottled, yellowish grey, with a poorly expressed yellowish brown band medially. Alternating grey and white portions of wing scales shaped as elongated spots, dots, and strokes on the lobes of all wings. Wingspan 12–15 mm	***A.bakingili***
–	Valves apically rounded. Anellus arms straight, not reaching the centre of gnathos	**31**
31	Anellus arms wide, distally much wider. Gnathos 3 × narrower than the width of anellus arms. Aedeagus without cornuti. Wings yellowish brown, with alternating elongated yellow-orange and dark brown spots on the first lobe of fore wing. Hind wings basally with an inclusion of dark spots of scales. Wingspan 12–14 mm	***A.rhaptica***
–	Anellus arms only slightly extended distally. Gnathos equal to anellus arms in width. Aedeagus with a cluster of needle-like cornuti distally. Wings greyish brown, basally darkened with dark-brown scales, medially with a wide pale yellow band, and distally with a wide dark brown band. Wingspan 14–15 mm	***A.lidiya***

## ﻿Discussion

The Mount Cameroon area hosts a remarkable diversity of Alucitidae. This paper adds nine newly described species and four other newly reported species, bringing the total count to 36 species. This comprises 40% of all 89 Alucitidae species known from the Afrotropical region, including 80 species listed in the Afromoths database (De Prins and De Prins 2023) and nine species described in this study. The extent of local diversity in this moth group is unprecedented, as only a few species of this group are known from any other locality in the region (Ustjuzhanin et al. 2018a, 2020a; De Prins and De Prins 2023).

Mount Cameroon is known to harbour high diversity in many taxa, including other Lepidoptera groups (e.g., Ballesteros-Mejia et al. 2013; Maicher et al. 2016; Przybyłowicz et al. 2019; Delabye et al. 2020, Mertens et al. 2021). The region’s exceptional species richness is often attributed to its location at the confluence of the Guinean and Congolian biogeographic regions, and a presence of diverse habitats along its elevational and precipitation gradients (Cable and Cheek 1998; Bergl et al. 2007; Hořák et al. 2019; Maicher et al. 2020a; Delabye et al. 2021; Doležal et al. 2022). Additionally, the area’s relatively high isolation further contributes to the unique ecological conditions (Ustjuzhanin et al. 2018a). However, the sheer magnitude of many-plumed moth species richness on Mount Cameroon surpasses expectations based on combinations of these exceptional factors in other Afrotropical localities. Despite this unique combination of conditions, it remains challenging to fully account for why Mount Cameroon exhibits such a substantial predominance of many-plumed moths compared to all other known sites in the Afrotropical region.

Notably, 24 of these species have been described solely from the Mount Cameroon area (as *A.ludmila* was already known from Nigeria and Ghana when it was described; Ustjuzhanin et al. 2018a), and the majority of them (except for *A.mischenini* and *A.zinovievi* recently reported from Liberia and Ghana, respectively; Ustjuzhanin et al. 2020b) are considered endemic to Cameroon and have not been recorded elsewhere. This level of endemism among the Alucitidae underscores the importance of Mount Cameroon as a vital refuge for specialised and unique insect taxa. While the region is already renowned for its rich endemic diversity of moths and butterflies (e.g., Larsen 2005; Sáfián and Tropek 2016; Przybyłowicz et al. 2019; Sáfián et al. 2019), as well as its endemic plant (Cable and Cheek 1998) and vertebrate (Fjeldså and Lovett 1997; Bergl et al. 2007) species, the unparalleled level of endemism of Alucitidae further highlights the area’s significance as a centre of microlepidopteran diversity in the Afrotropics.

The discovery of such a diverse and endemic locality on Mount Cameroon has exceeded expectations, despite the limited knowledge of Afrotropical microlepidoptera. The implications of this exceptional diversity extend beyond taxonomy, prompting future research into the evolutionary and ecological processes that have facilitated the development of this diverse moth community. The unique diversity and endemism of many-plumed moths in the Mount Cameroon area underscore the urgent need for efficient conservation of ecosystems and habitats in the region, especially considering previous conservation efforts that faced challenges in some parts (Ferenc et al. 2018). Collaborative efforts among researchers, conservationists, and local communities are crucial in preserving this treasure trove of biodiversity and securing the future not only for many-plumed moths.

## Supplementary Material

XML Treatment for Alucita pyrczi

XML Treatment for Alucita sroczki

XML Treatment for Alucita fako

XML Treatment for Alucita sedlaceki

XML Treatment for Alucita tonda

XML Treatment for Alucita erzayi

XML Treatment for Alucita sokolovi

XML Treatment for Alucita hirsuta

XML Treatment for Alucita potockyi

XML Treatment for Alucita agassizi

XML Treatment for Alucita dohertyi

XML Treatment for Alucita plumigera

XML Treatment for Alucita rhaptica

## ﻿Additional information

### Author ORCIDs

Peter Ustjuzhanin https://orcid.org/0000-0002-5222-2241

Vasily Kovtunovich https://orcid.org/0000-0001-5091-4263

Sylvain Delabye https://orcid.org/0000-0003-0911-9721

Vincent Maicher https://orcid.org/0000-0002-9147-3529

Szabolcs Sáfián https://orcid.org/0000-0002-0614-4203

Alexander Streltzov https://orcid.org/0000-0002-5658-8515

Robert Tropek https://orcid.org/0000-0001-7499-6259

### Data availability

All of the data that support the findings of this study are available in the main text.
